# Dual Role of Mitogen-Activated Protein Kinase 8 Interacting Protein-1 in Inflammasome and Pancreatic β-Cell Function

**DOI:** 10.3390/ijms24054990

**Published:** 2023-03-05

**Authors:** Rania Saeed, Abdul Khader Mohammed, Sarra E. Saleh, Mohammad M. Aboulwafa, Khaled M. Aboshanab, Jalal Taneera

**Affiliations:** 1 Department of Microbiology and Immunology, Faculty of Pharmacy, Ain Shams University, Cairo 11566, Egypt; 2 Sharjah Institute for Medical Research, University of Sharjah, Sharjah P.O. Box 27272, United Arab Emirates; 3 Faculty of Pharmacy, King Salman International University, Ras-Sudr, El Tor 8701301, Egypt; 4 Department of Basic Sciences, College of Medicine, University of Sharjah, Sharjah P.O. Box 27272, United Arab Emirates

**Keywords:** MAPK8IP1, inflammasome, regulation, pancreatic β-cell function, siRNA silencing, type 2 diabetes

## Abstract

Inflammasomes have been implicated in the pathogenesis of type 2 diabetes (T2D). However, their expression and functional importance in pancreatic β-cells remain largely unknown. Mitogen-activated protein kinase 8 interacting protein-1 (MAPK8IP1) is a scaffold protein that regulates JNK signaling and is involved in various cellular processes. The precise role of MAPK8IP1 in inflammasome activation in β-cells has not been defined. To address this gap in knowledge, we performed a set of bioinformatics, molecular, and functional experiments in human islets and INS-1 (832/13) cells. Using RNA-seq expression data, we mapped the expression pattern of proinflammatory and inflammasome-related genes (IRGs) in human pancreatic islets. Expression of *MAPK8IP1* in human islets was found to correlate positively with key IRGs, including the NOD-like receptor (NLR) family pyrin domain containing 3 (*NLRP3*), Gasdermin D (*GSDMD*) and Apoptosis-associated speck-like protein containing a CARD (*ASC*), but correlate inversely with Nuclear factor kappa *β*1 (*NF-κβ1*), Caspase-1 (*CASP-1*), Interleukin-18 (*IL-18*), Interleukin-1β (*IL-1β*) and Interleukin 6 (*IL-6*). Ablation of *Mapk8ip1* by siRNA in INS-1 cells down-regulated the basal expression levels of *Nlrp3*, NLR family CARD domain containing 4 (*Nlrc4*), NLR family CARD domain containing 1 (*Nlrp1*), *Casp1*, *Gsdmd*, *Il-1β*, *Il-18*, *Il-6*, *Asc*, and *Nf-κβ1* at the mRNA and/or protein level and decreased palmitic acid (PA)-induced inflammasome activation. Furthermore, *Mapk8ip1*-silened cells substantially reduced reactive oxygen species (ROS) generation and apoptosis in palmitic acid-stressed INS-1 cells. Nonetheless, silencing of *Mapk8ip1* failed to preserve β-cell function against inflammasome response. Taken together, these findings suggest that MAPK8IP1 is involved in regulating β-cells by multiple pathways.

## 1. Introduction

Type 2 diabetes (T2D) is an autoinflammatory metabolic disease caused by low-grade chronic inflammation due to overabundant nutrients and excessive metabolic stress [1,2,3]. The innate immune system appears to be primarily involved in evoking this metabolic inflammation (i.e., metaflammation) [4]. Inflammasomes are cytosolic multi-protein signaling complexes assembled upon recognition of various physiological and pathological stimuli. Inflammasome assembly triggers downstream signaling pathways that activate caspase-1, subsequently releasing pro-inflammatory cytokines (IL-1β and IL-18) and causing pyroptosis [5].

Pyroptosis is a programmed inflammatory cell death characterized by DNA fragmentation and the formation of pores in the plasma membrane resulting in the release of the cytosolic contents and the reinforcement of the immune response [6]. Although optimal inflammasome activation is favorable to the well-being of the host, aberrant inflammasome signaling can lead to an exaggerated innate immune response and the development of autoimmune and inflammatory disorders [7].

Several mechanistic studies have supported the involvement of inflammasome activation in the pathogenesis of T2D and its complications [8,9,10,11]. For example, a high glucose level was shown to induce NLRP3 [12,13]. Furthermore, NLRP3 and the secreted IL-1β were reported to be associated with insulin resistance [14,15], β-cell dysfunction, and cell death [16,17,18].

Interestingly, the protein expression of NLRP3, ASC, Caspase-1, IL-1β, and IL-18 were up-regulated in newly diagnosed T2D patients [19]. Another line of evidence has shown that inflammasome-mediated pyroptosis plays a key role in the occurrence and evolution of diabetes and its complications [20]. Furthermore, the inhibition or genetic deletion of inflammasome components has been found to improve glucose tolerance and insulin secretion and to reduce islet-cell apoptosis [8,10,18,21]. Thus, targeting inflammasome could be an early preventive strategy for diabetes and its complications [22,23]. However, the expression and function of inflammasome in pancreatic islets are still not well-characterized [24].

MAPK8IP1 (also known as Islet-Brain1 (IB1) protein or c-Jun N-terminal kinase (JNK) interacting protein-1 (JIP1)) is a scaffold protein that is highly expressed in the brain [25] and pancreatic β-cells [26]. There is accumulating evidence that MAPK8IP1 plays an essential role in β-cell survival and function. Although *MAPK8IP1* has been identified as a potential candidate gene for T2D [27], other studies have demonstrated that the loss of MAPK8IP1 function did not contribute to the development of diabetes [28,29]. Recently, we demonstrated that *MAPK8IP1* expression is reduced in human diabetic islets and that the silencing of *Mapk8ip1* in INS-1 cells impaired insulin secretion and reduced glucose uptake levels [30]. Furthermore, MAPK8IP1 has been reported to mediate the JNK signaling pathway [31], and the latter was implicated in inflammasome activation [32,33].

To our knowledge, no studies have investigated the role of MAPK8IP1 in pancreatic β-cell inflammasome regulation. Therefore, in this study, we aimed to explore the functional role of MAPK8IP1 in β-cell inflammasomes activation/regulation and its impact on β-cell survival and function.

## 2. Results

### 2.1. Expression Profiles of Pro-Inflammatory and Inflammasome-Related Genes (IRGs) in Human Pancreatic Islets

**Figure 1:**
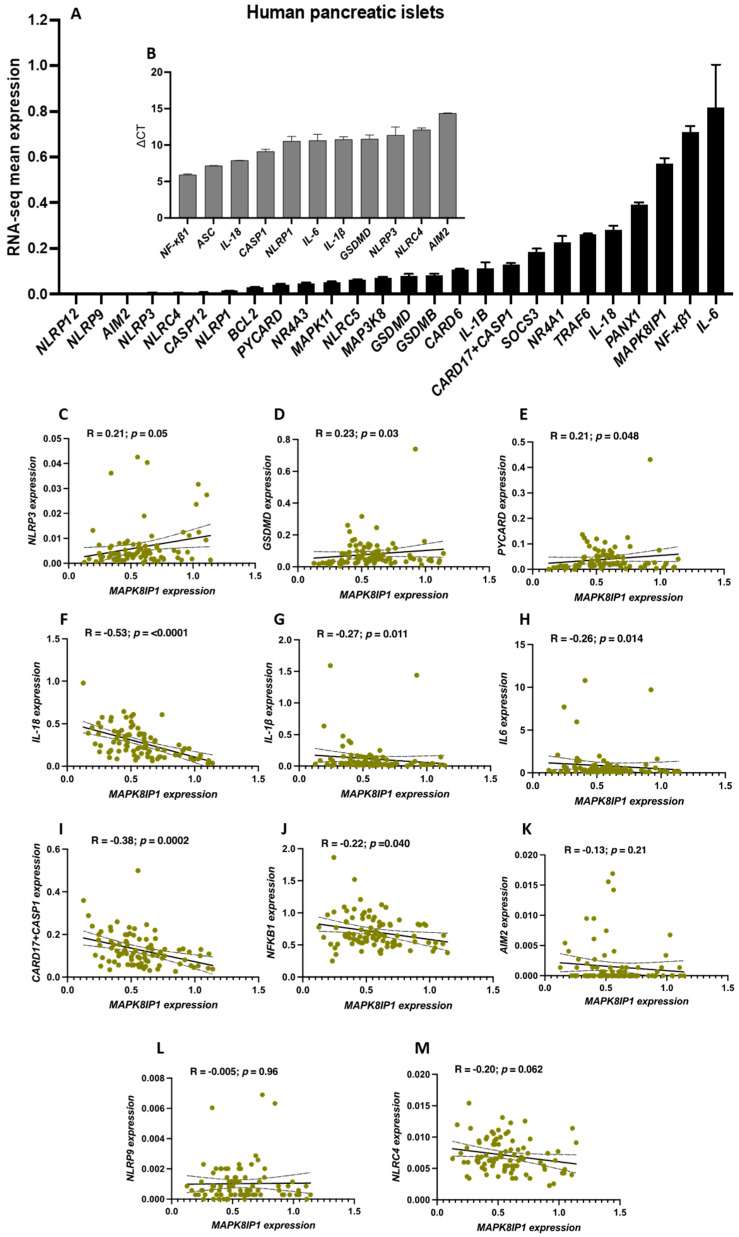
Expression profiles of IRGs in human pancreatic islets. (**A**) RNA-seq expression of *NLRP12*, *NLRP9*, *AIM2*, *NLRP3*, *NLRC4*, *CASP12*, *NLRP1*, *BCL2*, *PYCARD*, *NR4A3*, *MAPK11*, *NLRC5*, *MAP3K8*, *GSDMD*, *GSDMB*, *CARD6*, *IL-1β*, *CARD17*+*CASP1*, *SOCS3*, *NR4A1*, *TRAF6*, *IL-18*, *PANX1*, *MAPK8IP1*, *NF-κβ1*, and *IL-6* in non-diabetic human islets (*n* = 51) obtained from publicly available datasets. (**B**) qPCR expression analysis of *NF-κβ1*, *ASC*, *IL-18*, *CASP1*, *NLRP1*, *IL-6*, *IL-1β*, *GSDMD*, *NLRP3*, *NLRC4*, and *AIM2* in healthy human pancreatic islets obtained from a non-diabetic donor (*n* = 1; obtained from Prodo Lab, CA, USA). (**C–M**) Co-expression correlations of *MAPK8IP1* with (**C**) *NLRP3*, (**D**) *GSDMD*, (**E**) *PYCARD*, (**F**) *IL-18*, (**G**) *IL-1β*, (**H**) *IL-6*, (**I**) *CARD17*+*CASP1*, (**J**) *NF-κβ1*, (**K**) *AIM2*, (**L**) *NLRP9*, and (**M**) *NLRC4* using RNA-seq expression data from human pancreatic islets (*n* = 89). R and *p* values are indicated in the respective graphs. R: correlation coefficient; *p*: *p*-value. Bars above histograms represent the SDs of the mean values. The correlation analysis for Figure 1C–M was assessed using the nonparametric Spearman’s test.

Herein, we investigated the expression of several IRGs using published RNA-seq and qPCR expression analyses of human pancreatic islets. As is shown in Figure 1A, an RNA-seq expression analysis of 26 IRG genes showed that *IL-6*, *NF-κβ1*, *MAPK8IP1*, and pannexin1 (*PANX1*) were among the most highly expressed genes in the human islets, whereas NLR family pyrin domain containing 12 (*NLRP12*), NLR family pyrin domain containing 9 (*NLRP9*), absent in melanoma 2 (*AIM2*), *NLRP3*, and *NLRC4* were among the lowest expressed genes. For further confirmation, we evaluated the expression of 11 key genes involved in inflammasome activation in healthy human islets obtained from non-diabetic donors by qPCR. Based on ∆Ct values, an mRNA expression analysis revealed that *NF-κβ1*, *ASC*, and *IL-18* were among the more highly expressed genes in human islets, while *AIM2* and *NLRC4* were among those with the lowest expression (Figure 1B). Next, we correlated the expression of *MAPK8IP1* with IRGs in the pancreatic islets. As illustrated in Figure 1C–E, the expression of *MAPK8IP1* correlated positively (*p* < 0.05) with that of *NLRP3*, *GSDMD*, and *ASC* (also known as *PYCARD*), whereas it correlated inversely with that of *IL-18*, *IL-1β*, *IL-6*, *CARD17+CASP1*, and *NF-κβ1*. (Figure 1F–J). On the other hand, *AIM2*, *NLRP9*, and *NLRC4* showed no expression correlation with *MAPK8IP1* (Figure 1K–M). Together, these results reveal that the expression of *MAPK8IP1* is correlated with key IRGs in human islets.

### 2.2. Mapk8ip1 Silencing Influences IRGs in INS-1 (832/13) Cells

**Figure 2:**
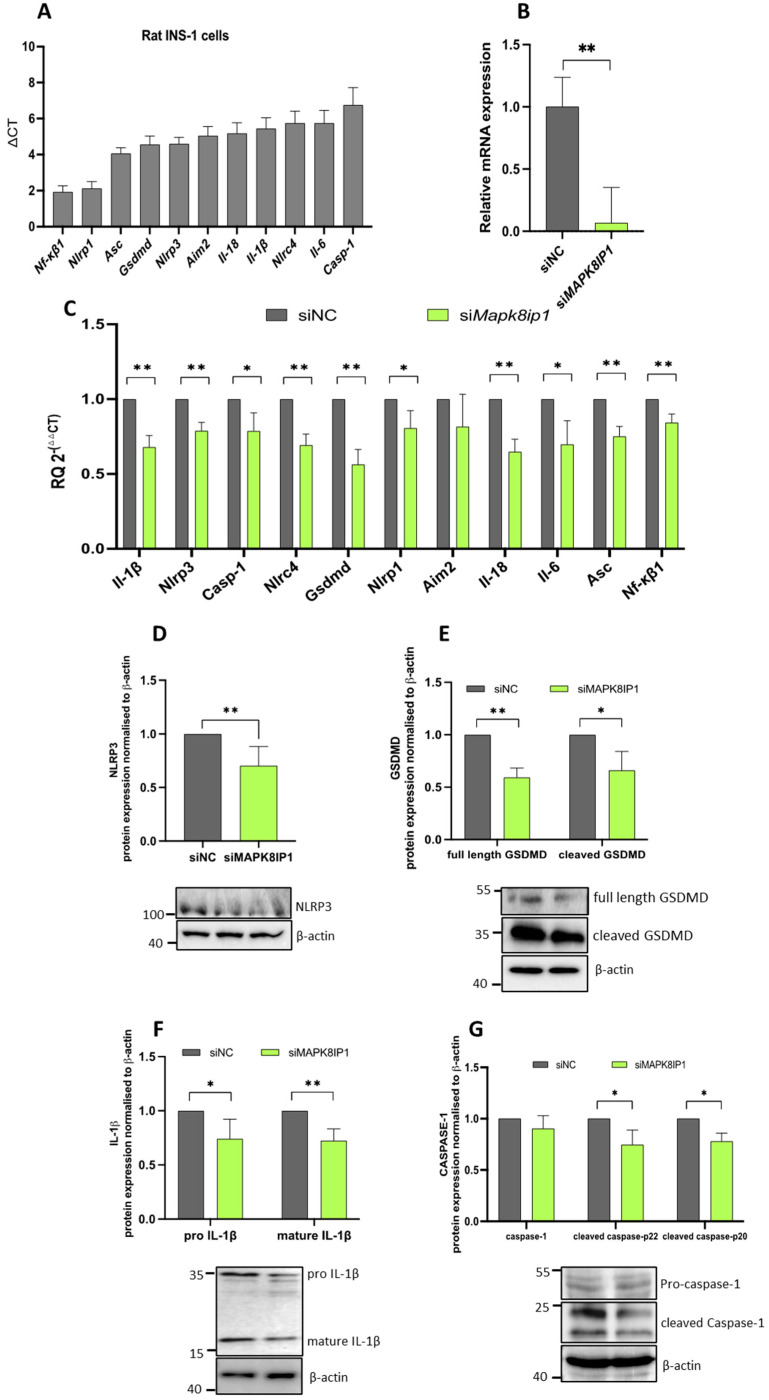
Impact of *Mapk8ip1* silencing on the expression of IRGs in INS-1 cells. (**A**) qPCR expression analysis of *Nf-κβ1*, *Nlrp1*, *Asc*, *Gsdmd*, *Nlrp3*, *Aim2*, *Il-18*, *Il-1β*, *Nlrc4*, *Il-6*, and *Casp1* in rat INS-1 (832/13) cells. (**B**) Silencing efficiency of *Mapk8ip1* mRNA expression as measured by qPCR 48 h post-transfection. Data were obtained from three independent experiments. (**C**) qPCR expression analysis of *Il-1β*, *Nlrp3*, *Casp1*, *Nlrc4*, *Gsdmd*, *Nlrp1*, *Aim2*, *Il-18*, *Il-6*, *Asc*, and *Nf-κβ1* in *Mapk8ip1*-silenced cells compared with those in negative control cells. Data were obtained from three independent experiments. (**D**–**G**) Western blot analyses of (**D**) NLRP3, (**E**) GSDMD (full-length and cleaved N-terminal fragment), (**F**) IL-1β (pro and mature IL-1β), and (**G**) CASPASE-1 (pro-caspase-1 and active cleaved caspase-1) relative to the endogenous control protein β-actin in the *Mapk8ip1*-silenced cells and negative control cells. Corresponding fold changes in the intensities of the Western blot bands are shown above each blot. Data were obtained from at least three independent experiments. * *p* < 0.05, ** *p* < 0.01, ns: not significant. Bars above histograms represent the SDs of the mean values. Statistical analyses were performed using the Student *t*-test.

Having established the expression profiles of these IRGs in human islets, we analyzed the expression profiles of 11 IRGs in rat INS-1 cells. As is shown in Figure 2A, *Nf-κβ1* and *Nlrp1* were highly expressed, whereas *Casp-1* and *Il-6* were expressed at lower levels compared with other genes. To further explore the impact of *Mapk8ip1* on the expression of IRGs, we silenced *Mapk8ip1* in the INS-1 cells using a pool of siRNA.

The results showed a significant reduction (*p* < 0.05) in *Mapk8ip1* mRNA levels (~82%) 48 h post-transfection compared with negative control cells (Figure 2B). Subsequently, we observed a significant decrease (*p* < 0.05) in the mRNA levels of the IRGs, including *Il-1β* (~32%), *Nlrp3* (~22%), *Casp1* (~22%), *Nlrc4* (~31%), *Gsdmd* (~44%), *Nlrp1* (~20%), *Il-18* (~35%), *Il-6* (~30%), *Asc* (~25%), and the transcriptional activator *Nf-κβ1* (~16%) compared with the control cells (Figure 2C). No significant alteration in the expression of *Aim2* was documented (Figure 2C). At the protein levels, a significant down-regulation (*p* < 0.05) was observed in NLRP3 (~30%; Figure 2D), GSDMD (full-length ~40% and cleaved N-terminal GSDMD ~34%; Figure 2E), and IL-1β (pro IL-1β ~26% and mature IL-1β ~28%; Figure 2F) in the *Mapk8ip1*-silenced cells versus controls. On the other hand, the protein expression of un-cleaved CASP-1 was not affected, whereas the expression level of cleaved caspase was reduced (~25%) (*p* < 0.05) (Figure 2G). The replicas of the full-length Western blot expressions after *Mapk8ip1* silencing are displayed in Supplementary Figures S1–S4.

Overall, these findings suggest that the silencing of *Mapk8ip1* leads to decreased expression levels of key IRGs at the mRNA and/or protein levels.

### 2.3. Inflammasome Activation Reduces Cell Viability and Alters the Expression of Pancreatic Β-Cell Function Genes

**Figure 3:**
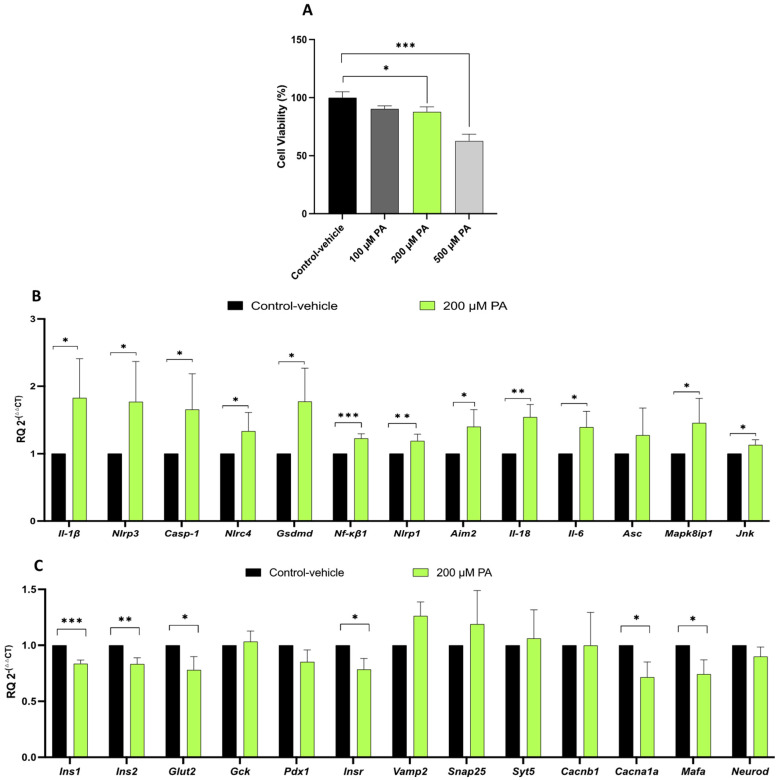
Impact of inflammasome activation on pancreatic β-cell function. INS-1 cells were cultured in the presence of LPS (1 μM) for 4 h and then stimulated with 200 μM PA–BSA for 24 h or cultured in the vehicle (control). (**A**) Cell viability assessment as determined by MTT assay showing the percentage of viable INS-1 cells following LPS/PA–BSA stimulation compared with the control cells. (**B**) qPCR expression analyses of key genes (*Il-1β*, *Nlrp3*, *Casp1*, *Nlrc4*, *Gsdmd*, *Nf-κβ1*, *Nlrp1*, *Aim2*, *Il-18*, *Il-6*, *Asc*, *Mapk8ip1*, and *Jnk*) involved in inflammasome activation in INS-1 cells following LPS/PA–BSA stimulation. (**C**) qPCR expression analyses of key genes (*Ins1*, *Ins2*, *Glut2*, *Gck*, *Pdx-1*, *Insr*, *Vamp2*, *Snap25*, *Syt5*, *Cacn1b*, *Cacna1a*, *Mafa*, and *Neurod*) involved in β-cell function in INS-1 cells following LPS/PA–BSA stimulation. Data were obtained from at least three independent experiments. * *p* < 0.05, ** *p* < 0.01, *** *p* < 0.001; ns: not significant. Bars above histograms represent the SDs of the mean values. Statistical analyses were performed using the Student *t*-test.

To investigate the potential of *Mapk8ip1* silencing to interfere with inflammasome activation, we initially assessed the impact of inflammasome activation on un-transfected INS-1 cells. Typically, inflammasome activation requires two signals. The first is for upregulating inflammasome components such as pro-IL-1β and NLRP3, which are brought by endotoxin lipopolysaccharide (LPS). The second signal is necessary to promote inflammasome assembly and is typically initiated by substances such as palmitic acid (PA). Therefore, we cultured INS-1 cells in the presence of LPS (1 μM) for 4 h and then stimulated them with various concentrations of palmitate conjugated to fatty acid-free bovine serum albumin (PA–BSA) for 24 h (100 μM, 200 μM, and 500 μM) [8]. Treatment with LPS/PA–BSA resulted in a significant reduction in cell viability (*p* < 0.05) at concentrations of 200 μM (~15%) and 500 μM (~37%), while no significant difference was observed at 100 μM PA–BSA compared with the vehicle control (Figure 3A). Based on these results, we selected a 1 μM LPS (4 h) followed by 200 μM PA–BSA stress regimen for further experiments, due to its impact on cell viability.

An expression analysis determined by qPCR revealed a substantial increase (*p  < * 0.05) in the mRNA levels of most of the genes involved in inflammasome assembly and activation at 200 μM PA–BSA compared with the vehicle control (Figure 3B). The up-regulated genes included *Il-1β*, *Nlrp3*, *Casp1*, *Nlrc4*, *Gsdmd*, *Nf-κβ1*, *Nlrp1*, *Aim2*, *Il-18*, *Il-6*, *Mapk8ip1*, and *Jnk* (Figure 3B). Notably, the expression of most β-cell function genes showed a significant down-regulation (*p* < 0.05) compared with the control (Figure 3C). The down-regulated genes included *Ins1*, *Ins2*, *Glut2*, *InsR*, *Cacna1a*, and *Mafa*.

These data indicate that exposing the cells to 1 μM LPS for 4 h, followed by 200 μM PA–BSA led to the up-regulation of most IRGs and the down-regulation of most β-cell function genes.

### 2.4. Expression Silencing of Mapk8ip1 Impairs β-Cell Inflammasome Activation in Stressed INS-1 Cells

**Figure 4:**
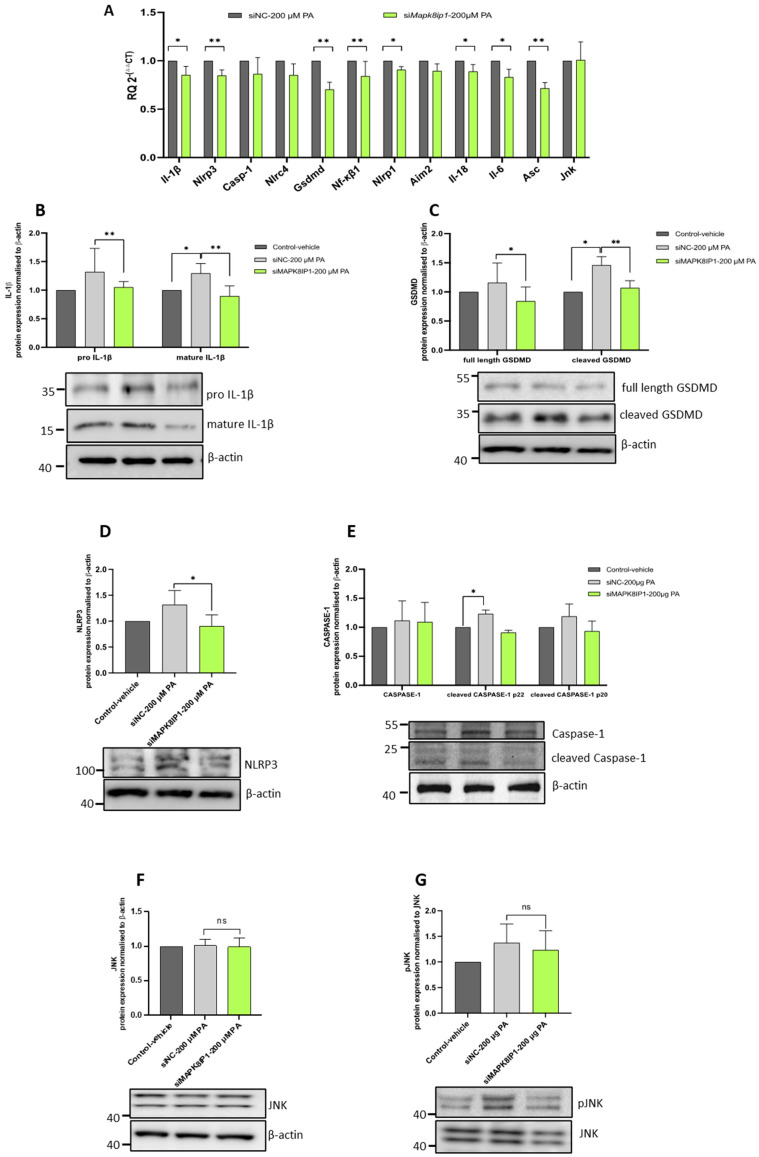
Impact of *Mapk8ip1* silencing on inflammasome activation. (**A**) qPCR expression analyses of *Il-1β*, *Nlrp3*, *Casp1*, *Nlrc4*, *Gsdmd*, *Nf-κβ1*, *Nlrp1*, *Aim2*, *Il-18*, *Il-6*, *Asc*, and *Jnk* in LPS/PA stressed *siMapk8ip1* cells compared with negative control cells. Data were obtained from three independent experiments. (**B**–**G**) Western blot analyses of (**B**) IL-1β (pro and mature IL-1β), (**C**) GSDMD (full-length and cleaved N-terminal fragments), (**D**) CASPASE-1 (pro-caspase-1 and active cleaved caspase-1), (**E**) NLRP3, (**F**) JNK, and (**G**) pJNK in *siMapk8ip1* cells compared with siNC cells in the presence of 1 μM LPS/200 μM PA–BSA or vehicle (control). All protein was normalized to β-actin; pJNK was normalized to JNK. Corresponding fold changes in the intensities of the Western blot bands are shown above each blot. Data were obtained from at least three independent experiments. * *p* < 0.05, ** *p* < 0.01, ns: not significant. Bars above histograms represent the SDs of the mean values. Statistical analyses were performed using the Student *t*-test.

Inflammasome assembly results in the cleavage of pro-caspase-1 and the formation of active caspase-1. Activated caspase-1 plays a crucial role in converting pro-IL-1β to mature IL-1β and cleaves GSDMD to form pores in the plasma membrane, which triggers pyroptosis [34]. In order to investigate and understand the impact of *Mapk8ip1* silencing on inflammasome activation, we analyzed the mRNA and protein expression of essential genes involved in inflammasome activation in LPS/PA–BSA stressed cells. An expression analysis using qPCR showed a significant reduction in the mRNA levels of *Il-1β* (~15%)*, Nlrp3* (~16%)*, Gsdmd* (~30%)*, Nf-κβ1* (~16%), *Nlrp1* (~10%), *Il-18* (~12%), *Il-6* (~17%), and *Asc* (~30%) in the *Mapk8ip1*-silenced LPS/PA–BSA stressed INS-1 cells compared with the negative controls (*p* < 0.05) (Figure 4A). However, the expression of *Casp-1*, *Nlrc4*, *Aim2*, and *Jnk* were not significantly affected (Figure 4A).

At the protein level, we observed a significant reduction in the expression and intracellular processing of pro-IL-1β to mature IL-1β (pro-IL-1β ~20% and mature IL-1β ~30%, *p* < 0.01) in the *Mapk8ip1*-silenced stressed INS-1 cells when compared with negative controls (Figure 4B). Additionally, the expression and cleavage of GSDMD and NLRP3 were also reduced (full-length GSDMD ~28%, *p* < 0.05; cleaved N-terminal GSDMD ~27%, *p* < 0.001; and NLRP3 ~31%, *p* < 0.05) (Figure 4C,D). Although the protein expression of activated CASP-1 and phosphorylated JNK showed a trend towards reduction in the *Mapk8ip1*-silenced stressed INS-1 cells, the data were not statistically significant (Figure 4E,G). The protein expression of JNK remained unaffected (Figure 4F). The replicas of the full-length Western blot expressions after *Mapk8ip1* silencing and LPS/PA–BSA treatment are displayed in Supplementary Figures S5–S7. Together, these findings strongly indicate that *Mapk8ip1* silencing impairs stimulation-induced inflammasome activation.

### 2.5. Expression Silencing of Mapk8ip1 Influences β-Cell Physiology

**Figure 5:**
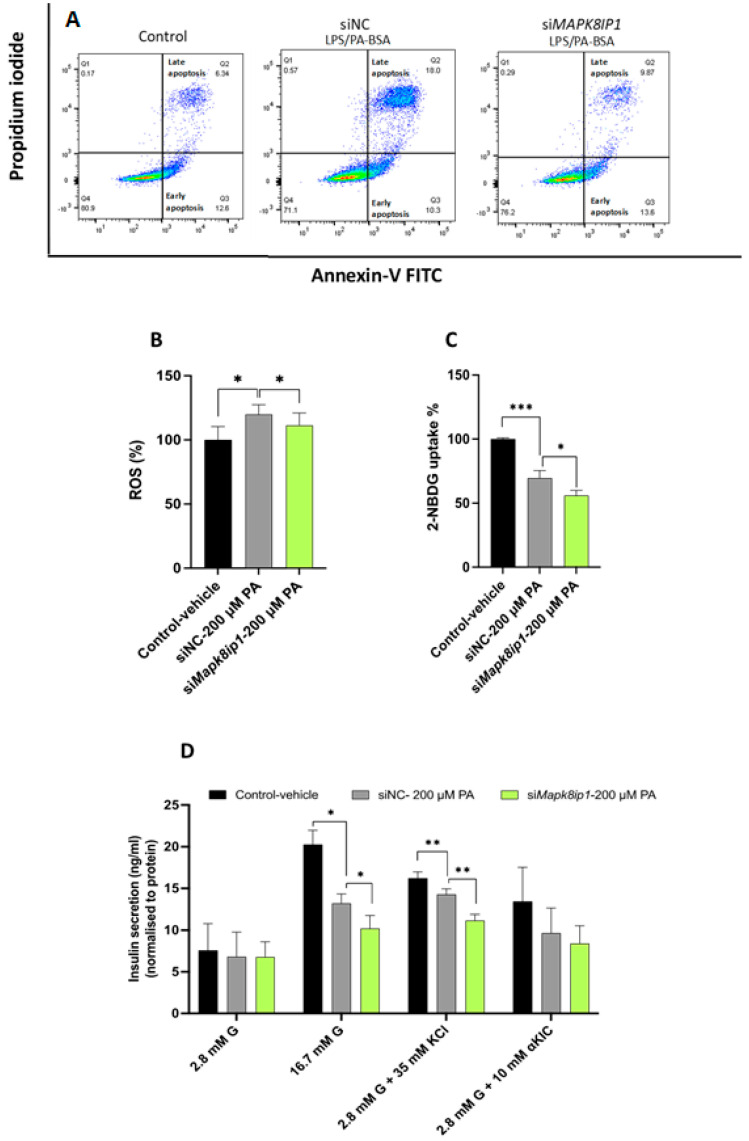
Effect of *Mapk8ip1* silencing on pancreatic β-cell physiology. (**A**) Analysis of apoptosis level in stressed *Mapk8ip1*-silenced INS-1 cells or negative controls in the absence (left panel) or presence (middle and right panel) of LPS/PA–BSA, as analyzed by flow cytometry. Data represent one experiment of three independent runs. (**B**) Detection of intracellular ROS levels by fluorescence intensity in *Mapk8ip1*-silenced cells compared with siNC cells in the presence of LPS/PA–BSA stimulation or vehicle (control). Data were obtained from three independent experiments. (**C**) Evaluation of glucose uptake efficiency in *Mapk8ip1*-silenced cells compared with siNC cells in the presence of LPS/PA–BSA stimulation or vehicle (control). Data were obtained from three independent experiments. (**D**) Normalized glucose-stimulated insulin secretion in response to low glucose (2.8 mM), high glucose (16.7 mM), and low glucose plus 35 mM potassium chloride (KCl) or 10 mM alpha-ketoisocaproic acid (α-KIC) in *Mapk8ip1*-silenced cells compared with siNC cells in the presence of LPS/PA–BSA or vehicle (control). Data were obtained from three independent experiments. * *p* < 0.05, ** *p* < 0.01, *** *p* < 0.001; ns: not significant. Bars represent mean ± SD. Statistical analyses were performed using the Student *t*-test.

Herein, we investigated the effects of *Mapk8ip1* silencing on apoptosis, ROS production, glucose uptake, and GSIS in INS-1 cells stressed with LPS/PA–BSA. The *Mapk8ip1*-silenced stressed cells exhibited a significant decrease (*p* < 0.05) in apoptosis rate (early and late apoptosis = 20% of the total number of cells) compared with the negative control silenced cells (~28%) (Figure 5A), indicating that the down-regulation of MAPK8IP1 counteracts, at least in part, the pro-apoptotic effect of PA. Additionally, we observed a significant reduction (*p* < 0.05) in intracellular ROS production and glucose uptake level in the LPS/PA–BSA-stimulated *Mapk8ip1*-silenced cells compared with the negative controls (Figure 5B,C).

Moreover, the *Mapk8ip1*-silenced stressed cells showed no change in their basal insulin secretion (2.8 mM glucose) but exhibited an impaired ability to augment the release of insulin at higher glucose concentrations (16.7 mM glucose) compared with the negative control cells (~18%, *p* < 0.05) (Figure 5D). Furthermore, insulin secretion stimulated by potassium chloride (KCl) was significantly reduced (~22%, *p* < 0.05) in the *Mapk8ip1*-silenced stressed cells compared with the negative controls. In contrast, no significant decrease in insulin secretion was observed upon alpha-ketoisocaproic acid (α-KIC) stimulation (Figure 5D).

**Figure 6:**
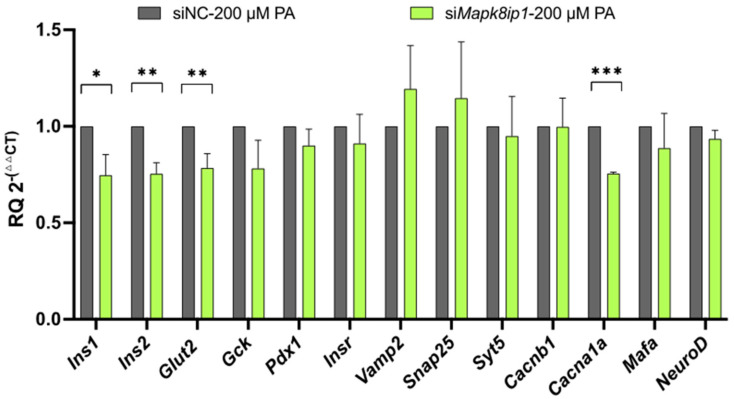
Impact of *Mapk8ip1* silencing and inflammasome activation on the expression of β-cell function genes. qPCR expression analysis of *Ins1*, *Ins2*, *Glut2*, *Gck*, *Pdx-1*, *Insr*, *Vamp2*, *Snap25*, *Syt5*, *Cacn1b*, *Cacna1*, *Mafa*, and *NeuroD* in LPS/PA-stimulated *siMapk8ip1* cells compared with negative control cells. Data were obtained from three independent experiments. * *p* < 0.05, ** *p* < 0.01, *** *p* < 0.001; ns: not significant. Bars above histograms represent the SDs of the mean values. Statistical analyses were performed using the Student *t*-test.

An analysis of the mRNA expression of β-cell function genes revealed significant reductions in *Ins1* (~25%), *Ins2* (~25%), *Glut2* (~22%), and *Cacna1a* (~22%) (*p* < 0.05) in the *Mapk8ip1*-silenced stressed cells compared with the negative control, whilst *Gck*, *Pdx-1*, *Insr*, *Vamp2*, *Snap25*, *Syt5*, *Cacnb*, *Mafa*, and *NeuroD* remained unaffected (Figure 6A). These findings indicate that silencing *Mapk8ip1* reduced reactive oxygen species (ROS) generation, apoptosis, GSIS, and glucose uptake in stressed INS-1 cells and altered the expression of several pancreatic β-cell function genes.

### 2.6. Mapk8ip1 Silencing Reduces GSDMD Expression in INS-1 Cells

**Figure 7:**
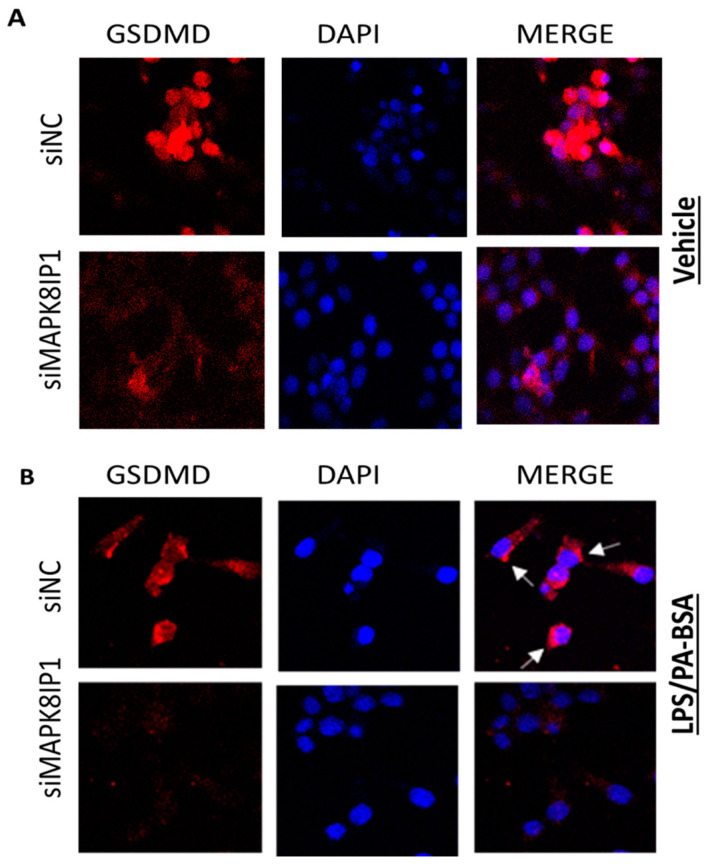
Confocal microscopy images showing the expression of GSDMD. siNC (upper panel) and *siMapk8ip1*-silenced cells (lower panel) were either (**A**) left untreated (vehicle) or (**B**) incubated with LPS (1 μM) and stimulated with PA–BSA (200 μM). DAPI was used to stain the nuclei. Arrows indicate pyroptotic bodies in the plasma membrane where GSDMD accumulated after the treatment of the INS-1 cells with LPS/PA–BSA.

GSDMD is a component of the inflammasome responsible for forming membrane pores and the induction of pyroptosis. When stimulated, caspase-1 cleaves GSDMD, which releases the N-terminal p30 domain. This domain binds to phospholipids on the plasma membrane, resulting in the formation of large oligomeric pores that facilitate the release of cellular contents and mature IL-1β [34]. Therefore, we sought to examine the expression of GSDMD in control and *Mapk8ip1*-silenced INS-1 cells via confocal microscopy. As is shown in Figure 7A, the confocal microscopic analysis confirmed the expression of GSDMD in unstimulated INS-1 cells (Figure 7A, upper panel).

Upon stimulation with LPS/PA–BSA, GSDMD translocates towards the plasma membranes, resulting in the appearance of pyroptotic bodies in the membranes where GSDMD accumulates (Figure 7B, upper panel, indicated by white arrows). In contrast, *Mapk8ip1* silencing led to a decrease in the expression of GSDMD in both the untreated (Figure 7A, lower panel) and the treated cells (Figure 7B, lower panel). Furthermore, the accumulation of activated GSDMD in the plasma membrane was also reduced in the *Mapk8ip1*-silenced cells. Thus, these findings suggest that *Mapk8ip1* silencing reduces the expression of GSDMD in both stressed and unstressed INS-1 cells.

## 3. Discussion

In this study, we specifically evaluated the regulatory role of MAPK8IP1 in inflammasome activation in pancreatic β-cells. Our data described the expression profiles of several IRGs in human islets and INS-1 cells and identified significant correlations with *MAPK8IP1*. The study demonstrated that reduced expression of *Mapk8ip1* in INS-1 cells decreased the expression of IRGs, such as *Nlrp3*, *Nlrp1*, and *Nlrc4*, and impaired stimulation-induced inflammasome activation. Furthermore, the silencing of *Mapk8ip1* reduced ROS generation and attenuated stress-induced apoptosis. Despite the observed down-regulation of the inflammatory pathway in stressed INS-1 cells, the silencing of *Mapk8ip1* failed to restore β-cell function, as evidenced by the decreased insulin secretion, glucose uptake, and altered expression of several pancreatic β-cell function genes. These findings suggest that MAPK8IP1 plays an important role in inflammasome regulation.

It is well-documented that upon activation, NLR genes form a complex with the adaptor protein, ASC, which facilitates the activation of pro-caspase-1, forming active caspase-1 p20 tetramer. Activated caspase-1 is responsible for the maturation of the active forms of proinflammatory cytokines IL-18 and IL-1β [34] in addition to the cleaving of GSDMD to trigger pyroptosis [34]. Our findings revealed that the reduction in the expression of NLR genes in the *Mapk8ip1*-silenced cells was associated with reduced expression levels of *Asc*, *Casp-1*, and the three caspase-substrates *Il-1β*, *Il-18*, and *Gsdmd*. Moreover, we also noticed reduced expression levels of *Nf-κβ1* and *Il-6*. NF-κβ1 is the transcriptional activator of NLRP3 and pro-IL-1β [11,35], while IL-6 is a downstream effector of IL-1β [36]. In line with previous findings [10], we hypothesize that the reduced *Il-6* and Nf-κβ1 levels in the *Mapk8ip1*-silenced cells might reflect impaired IL-1β bioactivity or inflammasome activity.

Several IRGs showed decreased expression at the mRNA and/or protein levels when the *Mapk8ip1*-silenced cells were stressed with LPS/PA–BSA. Among these genes are the cleaved GSDMD N-terminal fragment and mature IL-1β, which are essential effectors of inflammasome activation and mediators of the inflammatory cascade [34]. Furthermore, the accumulation of activated GSDMD in the plasma membrane of the LPS/PA–BSA-treated cells was also reduced in the *Mapk8ip1*-silenced cells, as was shown by the confocal microscopy. Hence, it seems that *Mapk8ip1* silencing influences the expression of genes involved in pyroptosis.

It has been stated that MAPK8IP1 protein functions as a regulator of the JNK signal transduction pathway [28,31]. Phosphorylation of JNK is a critical step for NLRP3 assembly [32] and ASC transcriptional regulation [33]. Therefore, it is conceivable that the impact of MAPK8IP1 on inflammasome activation might result from a MAPK8IP1-induced modulation of JNK. In support of this, our data revealed that *Mapk8ip1*-silenced cells exhibited a trend towards reduced stress-induced JNK activation following LPS/PA–BSA stimulation. Consistent with these findings, several studies have demonstrated the requirement of the MAPK8IP1 scaffold protein for stress-induced JNK activation [28,37]. On the other hand, the observed impairment in inflammasome activation following *Mapk8ip1* silencing might be attributed to the down-regulation of different inflammasome subtypes, such as NLRP1 or NLRC4, which are not substantially affected by JNK [38,39]. Future studies are thus required to fully define the mechanism of inflammasome regulation via the JNK–MAPK8IP1 signaling axis, possibly by testing different JNK isoform knockdowns and various stressors [40].

Circulating free fatty acids have been linked to the pathogenesis of T2D and metabolic inflammation in various tissues in the body [41,42]. Previous studies have suggested that free fatty acids may activate toll-like receptors (TLR), leading to inflammasome activation and the production of proinflammatory cytokines [43,44]. It has been reported that IL-1β elevates the risk for T2D by inducing insulin resistance [14] and increasing β-cell apoptosis [16]. Our findings confirm that fatty acids (e.g., PA) exert their pro-inflammatory effects by activating inflammasomes and causing IL-1β release in β-cells, exacerbating cell death. *Mapk8ip1* silencing down-regulated inflammasome activation and decreased PA-induced cell death, indicating that the inflammasome signaling axis is involved in PA-induced β-cell death. Thus, an inflammasome antagonist could be a promising therapy for T2D [23].

Similarly, ROS have been identified as one of the early triggers of inflammasome activation [45,46], and they play a pivotal role in promoting β-cell dysfunction [47,48]. PA is a potent inducer of ROS [8,49,50] and contributes to inflammasome activation and β-cell loss [8,44,51]. Our results confirm that PA-induced inflammasome activation is associated with increased ROS generation. However, the silencing of *Mapk8ip1* was found to attenuate ROS production in PA-stressed INS-1 cells. Therefore, the reduced ROS generation following *Mapk8ip1* silencing could contribute to the down-regulation of inflammasome activation, as indicated by decreased NLRP3, IL-1β and GSDMD expression.

Despite the evidence supporting the critical role of MAPK8IP1 in regulating inflammasome activation, the translation of such findings to β-cell function has yielded disappointing outcomes with respect to insulin secretion, glucose uptake, and the expression of key β-cell functional genes. A previous study reported that MAPK8IP1 is required for GLUT2 expression and is a candidate for T2D [27]. In contrast, Whitemarsh et al. demonstrated that the loss of MAPK8IP1 function does not directly cause diabetes [28]. Our results support the notion that MAPK8IP1 is involved in regulating insulin secretion.

While it is undeniable that there is a reduction in insulin secretion in both unstressed [30] and stressed *Mapk8ip1*-silenced INS-1 cells, we noticed an improvement in the *siMapk8ip1*-induced decrease in GSIS under stress. *Mapk8ip1*-silenced stressed INS-1 cells showed ~18%, ~22%, and 12% reductions in GSIS with 16.7 mM glucose, KCL, and α-KIC stimulation, respectively, while unstressed *siMapk8ip1* cells showed ~30%, ~33%, and ~40% reductions in GSIS with 16.7 mM glucose, KCL, and α-KIC stimulation, respectively [30].

It is worth noting that this study has certain limitations. Although caspase-1 is the primary canonical intracellular enzyme responsible for the maturation of proIL-1β and GSDMD cleavage, there is also a noncanonical pathway involving caspase-4/-5/-11 that may be responsible for the processing of IL-1β and which occurs independently of inflammasome assembly [33,52]. Thus, it is necessary to assess the contribution of other caspases to the inflammatory pathway.

## 4. Materials and Methods

### 4.1. RNA-Seq Expression Data from Human Pancreatic Islets

A publicly available RNA-seq transcriptomic dataset (GSE50398) was retrieved from NCBI’s Gene Expression Omnibus (GEO; “https://www.ncbi.nlm.nih.gov/bioproject/?term=GSE50398 (accessed on 1 February 2020)” [53]. The expression data were obtained from 89 cadaver donors (European ancestry). Of these, 45 were non-diabetic/normoglycemic donors (HbA1c < 6%) and 33 were diabetic/hyperglycemic (6% ≤ HbA1c < 6.5%).

### 4.2. Culturing of INS-1 Cell Line and Palmitic Acid Treatment

Rat insulinoma INS-1 (832/13) cells (Research Resource Identifier RRID:CVCL_7226) were kindly provided by Dr. C. B. Newgaard of Duke University, USA [54]. As was previously described, rat insulinoma INS-1 (832/13) cells were cultured in RPMI-1640 medium [55]. For the palmitic acid treatment, PA (Sigma-Aldrich, Darmstadt, Germany) was dissolved in 0.1 mmol/l NaOH at 70 °C for 30 min and then conjugated to appropriate amounts of fatty acid-free bovine serum albumin (BSA) at 60 °C for 30 min at a molar ratio of 5:1 [56]. Next, the palmitate–BSA (PA–BSA) conjugate was diluted in serum-free RPMI-1640 medium supplemented with 1% BSA to final concentrations of 100 μmol/L, 200 μmol/L, and 500 μmol/L palmitic acid and added to the cells. For the control, we used the same concentration of vehicle (100 mM NaOH–BSA) in RPMI medium.

The INS-1 cells were cultured in 24-well plates (2.0 × 10^5^ cells/well) for inflammasome activation until they reached 80% confluence. The cells were then stimulated with LPS (1 μg/mL) (LPS, Sigma-Aldrich L4391, from *Escherichia coli* 0111:B4) for four hours, then incubated with 200 μM PA–BSA [24,57]. Following incubation, the cells were used for functional validation assays.

### 4.3. siRNA Transfection

The INS-1 (832/13) cells were seeded in a 24-well plate (200,000 cells/well) in a complete RPMI 1640 medium and transfected with two sets of siRNA sequences for MAPK8IP1 (s137914 and s137915) (Thermo Fisher Scientific, Waltham, MA, USA) or scramble negative control siRNA, as previously described [55]. To test the effect of inflammasome activation, 24 h post-transfection, the cells were pretreated with LPS for 4 h and incubated with 200 μM PA–BSA for 24 h. A separate group of transfected cells for the control was similarly treated with vehicle (NaOH in serum-free RPMI containing 1% BSA). Following incubation, the mRNA from the treated transfected cells was isolated for further analysis.

### 4.4. RNA Extraction and qRT-PCR

A High-Capacity cDNA Reverse Transcription Kit (Thermo Fisher Scientific, Waltham, MA, USA) was used to synthesize complementary DNA (cDNA) from the extracted RNA. An expression analysis of the key genes involved in β-cell function from the transfected and non-transfected treated cells was assessed with qPCR using TaqMan gene expression assays through the use of gene-specific primer probes for *Mapk8ip1* (Rn00587215_m1), *Glut2* (Rn00563565_m1), *Ins1* (Rn02121433_g1), *Ins2* (Rn01774648_g1), *Pdx1* (Rn00755591_m1), *Insr* (Rn00690703_m1), *Gck* (Rn00561265_m1), and Rat *Hprt1* (Rn01527840_m1). A SYBR green gene expression analysis for several β-cell function genes was conducted using the corresponding primers (Table 1). An expression analysis of the principle genes implicated in β-cell inflammasome assembly/activation was assessed using qRT-PCR via SYBR green gene expression assays using the primers listed in Table 1. Rat Hprt1 was used as an endogenous control for normalizing the expression of the target mRNA. Relative gene expression was assessed using the 2^−ΔΔCt^ method. All qPCR reactions were run in 96-well plates in triplicate using the QuantStudio 3 qPCR system (Applied Biosystems, Waltham, MA, USA).

### 4.5. Insulin Secretion Assay

GSIS measurements were performed 48 h post-transfection, as previously described [58]. First, INS-1 (832/13) β-cells were incubated in pre-warmed secretion assay buffer (SAB) with 2.8 mM glucose for 2 h. The cells were then stimulated with SAB containing either 2.8 mM glucose, 16.7 mM glucose, 2.8 mM glucose plus 10 mM α-KIC, or 35 mM KCl for 1 h. Next, the amount of secreted insulin was determined using the rat insulin ELISA kit (Mercodia, Uppsala, Sweden) and normalized to the total amount of protein.

### 4.6. Western Blot Analysis

To detect activated inflammasome proteins, INS-1 cells were stimulated with 1 µL LPS/200 μM PA–BSA for 4 h [24,59]. Total protein extraction was performed using ice-cold NP-40 (1.0% NP-40, 150 mM NaCl, 50 mM Tris-Cl, pH 8.0) lysis buffer containing a protease inhibitor cocktail (Thermo Fisher Scientific, Waltham, MA, USA). A Western blot analysis was performed as previously described [58] with the following antibodies: MAPK8IP1 (anti-rabbit; 1:1000, #Ab24449, Abcam, Cambridge, UK), NLRP3 (anti-rabbit; 1:1000, #A12694, Abclonal, Woburn, MA, USA), CASPASE-1 (anti-rabbit; 1:1000, #A0964, Abclonal, Woburn, MA, USA), IL-1β (anti-rabbit; 1:1000, #A162888, Abclonal, USA), GSDMD (anti-rabbit; 1:1000, #A10164, Abclonal, USA), JNK (anti-rabbit; 1:1000, #A48567, Abclonal, USA), pJNK (anti-rabbit; 1:1000, #AP0631, Abclonal, USA), β-actin (anti-mouse, 1:1000, #A5441, Sigma-Aldrich, Darmstadt, Germany), and secondary anti-mouse (#7076S) or anti-rabbit (#7074S, from Cell Signaling Technology, Danvers, MA, USA). Chemiluminescence was detected using the Clarity ECL substrate kit (Bio-Rad, Hercules, CA, USA). β-actin was used as an endogenous control.

### 4.7. Apoptosis Assay

The transfected and non-transfected cells were cultured in RPMI medium in the presence of vehicle (control) or 1 µL LPS followed by 200 μM PA–BSA, as mentioned earlier. Following 24 h incubation, the cells were re-suspended in 500 μL of Annexin-V (1X) Binding Buffer (BD Biosciences, San Jose, CA, USA) and then stained with 2 μL of Annexin V-FITC and 2 μL of Propidium Iodide (PI) (15 min) in the dark. The cells were analyzed using a BD FACS Aria III flow cytometer (Becton Dickinson, Biosciences, Franklin Lakes, NJ, USA).

### 4.8. Cell Viability Assay

An MTT colorimetric assay (Sigma-Aldrich, Saint Louis, MO, USA) was used to assess cell viability. In brief, transfected and non-transfected INS-1 (832/13) cells, seeded in 96-well plates (20 × 10^4^/well), were cultured in RPMI medium in the presence of vehicle (control) or 1 µL LPS followed by PA–BSA for 24 h, as mentioned earlier. An aliquot (10 µL) of MTT solution was added to each well and incubated at 37 °C for 2 h. The formed MTT formazan crystals were dissolved in 100 μL DMSO and the absorbance was measured using a microplate reader at an optical density of 570 nm. The cell viability percentage was calculated.

### 4.9. Glucose Uptake

The glucose uptake in the cultured cells was assessed using 2-NBDG (Invitrogen #N13195, Carlsbad, CA, USA). Briefly, the transfected and non-transfected INS-1 cells were cultured in the presence of vehicle (control) or 1 µL LPS followed by 200 μM PA–BSA for 24 h, as mentioned earlier. Forty-eight hours post-transfection, 100 µM of 2-NBDG was added to each well and incubated at 37 °C for one hour, as previously described [58]. The cells were then trypsinized and analyzed using flow cytometry (BD FACS AriaTM III flow cytometer, San Jose, CA, USA).

### 4.10. ROS generation

According to the manufacturer’s instructions, the intracellular generation of ROS was detected using a ROS-Glo H_2_O_2_ assay kit (Cat #G8820, Promega, Madison, WI, USA). Briefly, the transfected and non-transfected INS-1 cells were treated with 1 μg/mL LPS followed by 200 μM PA–BSA or vehicle (control) for 24 h. Forty-eight hours post-transfection, the cells were incubated with H_2_O_2_ substrate for 3 h at 37 °C. ROS-Glo detection reagent was added, and the cells were incubated for 20 min at room temperature. The relative luminescence was then detected using a plate reader [58].

### 4.11. Immunofluorescence Assay

The transfected INS-1 cells were plated on glass coverslips and treated with 1 μg/mL LPS/200 μM PA–BSA or vehicle (control). The cells were then fixed using 4% paraformaldehyde for 15 min at room temperature and permeabilized with 0.2% Triton X-100 in phosphate-buffered saline (PBS) for 5 min. Glass coverslips were blocked using 1% Triton X-100 + 2% BSA in phosphate-buffered saline (PBS) for 1 h followed by overnight incubation with a primary antibody against GSDMD (anti-rabbit; 1:1000, #A10164, Abclonal, USA). The cells were washed 3 times with 0.1% Triton X-100 in phosphate-buffered saline (PBS) for 5 min and then labeled with respective secondary antibodies tagged with Alexa 488 for 1.5 h. The coverslips were mounted on slides using mounting media with DAPI (Invitrogen, Carlsbad, CA, USA) to stain the nucleus. The slides were then observed under a confocal microscope (A1R Confocal Laser Microscope System, Nikon Inc., Tokyo, Japan).

### 4.12. Statistical Analysis

A Student *t*-test or a nonparametric Mann-Whitney test was used for the differential expression analysis between the diabetic and non-diabetic donors. The correlation between the variables was calculated using a nonparametric Spearman’s test. All statistical analyses were performed using GraphPad Prism (version 8.0.0, “www.graphpad.com (accessed on 1 February 2020)”).

## 5. Conclusions

In summary, our data suggest that MAPK8IP1 could be an important mediator of β-cell inflammasome. However, despite our promising mechanistic studies identifying MAPK8IP1 as an inflammasome regulator, the therapeutic potential of using MAPK8IP1 to ameliorate T2D seems to be impeded by its role in β-cell function and insulin secretion. This indicates that MAPK8IP1 is involved in multiple pathways that regulate pancreatic β-cell function.

## Figures and Tables

**Table 1:** SYBR green primer sequence used in this study.

No.	Gene/Symbol	Forward Primers (5′-3′)	Reverse Primers (5′-3′)
1	*Nlrp3*	GCCTCATCCGAAAGAAGTTGC	TGGCCTCAGAGAAACCTAGGA
2	*Casp-1*	GTGGTTCCCTCAAGTTTTGC	GTGCTGCAGATAATGAGGGCA
3	*Il-1β*	CAGCAATGGTCGGGACATAG0	AGACTGCCCATTCTCGACAAG
4	*IL-18*	ATTCTTTCAGAAACGTGTGCCAG	ATCCCCATTTTCATCCTTCC
5	*Gsdmd*	CGGGAGTGGTCAAGAATGTG	CATGAGCTTGAGAGTTTCCTGC
6	*Nlrp1*	GCCCTCTGCCTGAAATACCTT	CAGGGTCCTTCTTTGGCAGAT
7	*Nlrc4*	ACTCCATCAGCAAACCAACC	CCTCGATTTCTGGGCAGTTCT
8	*NF-κβ1*	CCACACTGTAAACCAAAGCCC	GGAAGGCCTCGAATGACATCA
9	*Aim2*	TCACCAGTTCCTCAGTTGTGG	GCACGGCAGAGTTTTCAGTTT
10	*Asc*	AAGGACAGTACCAGGCAGTTC	CCAAGTAGGGCTGTGTTTGC
11	*Il-6*	GCCTATTGAAAATCTGCTCTGG	ATTGCTCTGAATGACTCTGG
12	*Hprt*	TTGTGTCATCAGCGAAAGTGG	CACAGGACTAGAACGTCTGCT
13	*Mafa*	GAGGAGGAGCGCAAGATCGG	AGCAAAAGTTTCGTGCTGTCAA
14	*NeuroD1*	CCCTAACTGATTGCACCAGC	TGCAGGGTAGTGCATGGTAA
15	*Syt5*	CACCTGACCCCAGATCCTTT	GAGTGGTACTGGAAGTCGGA
16	*Snap25*	GGCGTTTGCTGAATGACAAC	CAGAGCCTGACACCCTAAGA
17	*Cacna1a*	CTAGCCCTGCCAAGATCGG	ACGATAAGGCTGTTCTCGG
18	*Cacnb1*	CTTTACCCCAGCAACCACCC	GTCCACACACGAGTCTCCTG
19	*Vamp2*	TGGTGGACATCATGAGGGTG	GCTTGGCTGCACTTGTTTCA
20	*Jnk*	TCCAGTTCTCGTACCCGCTA	AGCATGGCGTGACACAGTA

## Data Availability

Publicly available datasets were analyzed in this study. This data can be found here: “https://www.ncbi.nlm.nih.gov/geo/query/acc.cgi?acc=GSE50398) (accessed on 1 February 2020)”/accession number: GSE50398.

## Supplementary Materials

The following supporting information can be downloaded at: https://www.mdpi.com/article/10.3390/ijms24054990/s1, Figures S1–S4 (replicas 1–4): The full-length Western blot expressions after *Mapk8ip1* silencing, Figures S5–S7 (replicas 1–3): The full-length Western blot expressions showing the impact of *Mapk8ip1* silencing on inflammasome activation using LPS/PA–BSA.
