# Supplementary material for: Dual Role of Mitogen-Activated Protein Kinase 8 Interacting Protein-1 in Inflammasome and Pancreatic β-Cell Function
Source: Int J Mol Sci. 2023 Mar 5;24(5):4990. doi: 10.3390/ijms24054990 (PMC10002854; doi:10.3390/ijms24054990)
Supplement: Supplementary file 1 [file ijms-24-04990-s001.zip › ijms-2204625-supplementary.pdf]

Supplementary Figures

Figure S1 (replicate 1):

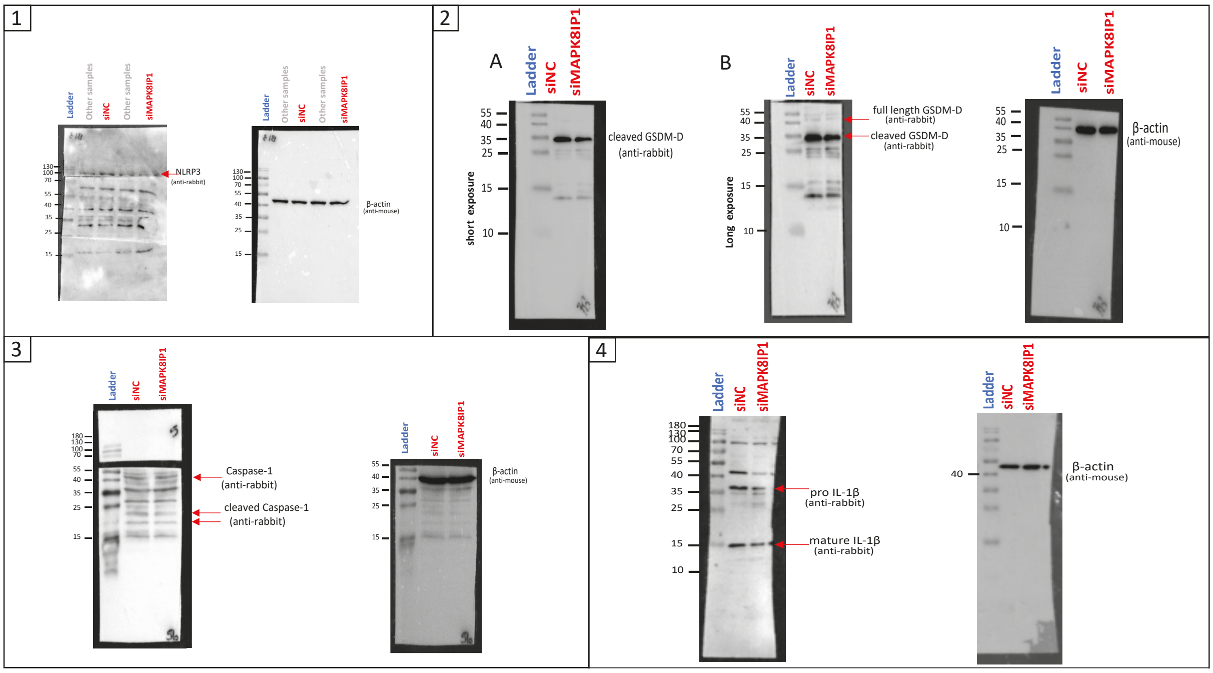

Figure S1. (replicate 1) The full-length western blot expressions after *Mapk8ip1* silencing. Western blot was conducted for **(1)** NLRP3, **(2)** GSDM-D (full length and cleaved N-terminal fragment) after (A) short exposure time and (B) long exposure time, **(3)** CASPASE-1 (pro-caspase-1 and active cleaved caspase-1) and **(4)** IL-1β (pro and mature IL-1β). β-actin was used as endogenous control. The cropped blots from membranes 1, 2, 3 and 4 were used as representative figures in the main article.

Figure S2 (replicate 2):

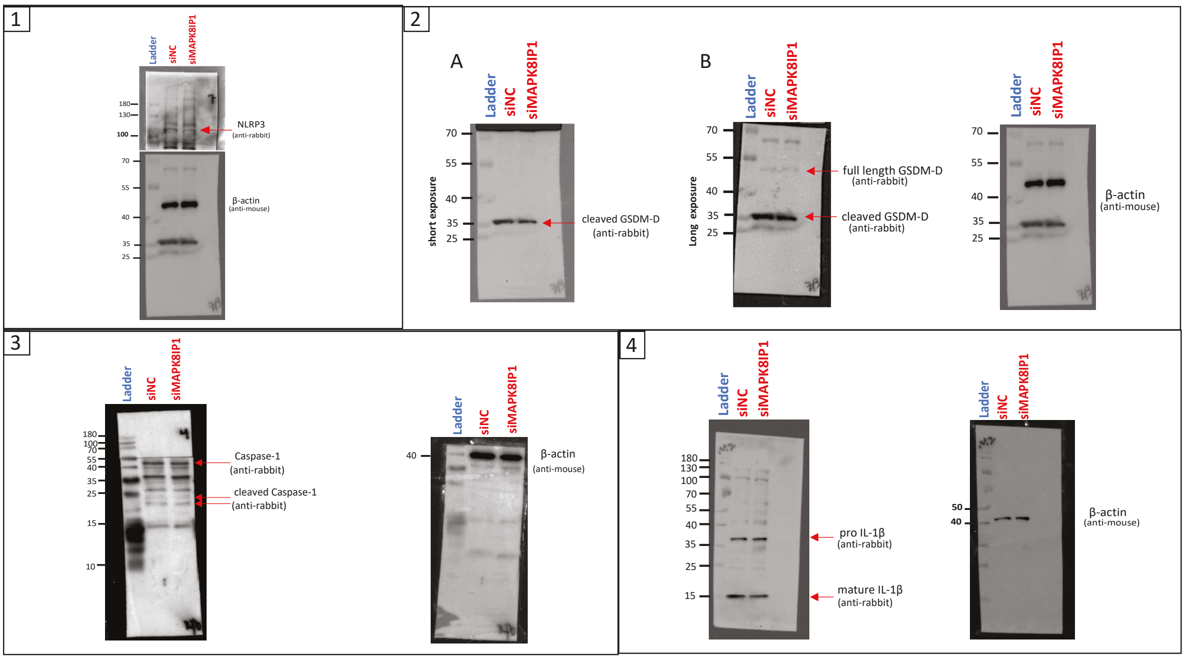

Figure S2. (replicate 2) The full-length western blot expressions after *Mapk8ip1* silencing. Western blot was conducted for **(1)** NLRP3, **(2)** GSDM-D (full length and cleaved N-terminal fragment) after (A) short exposure time and (B) long exposure time, **(3)** CASPASE-1 (pro-caspase-1 and active cleaved caspase-1) and (**4)** IL-1β (pro and mature IL-1β). β-actin was used as endogenous control.

Figure S3 (replicate 3):

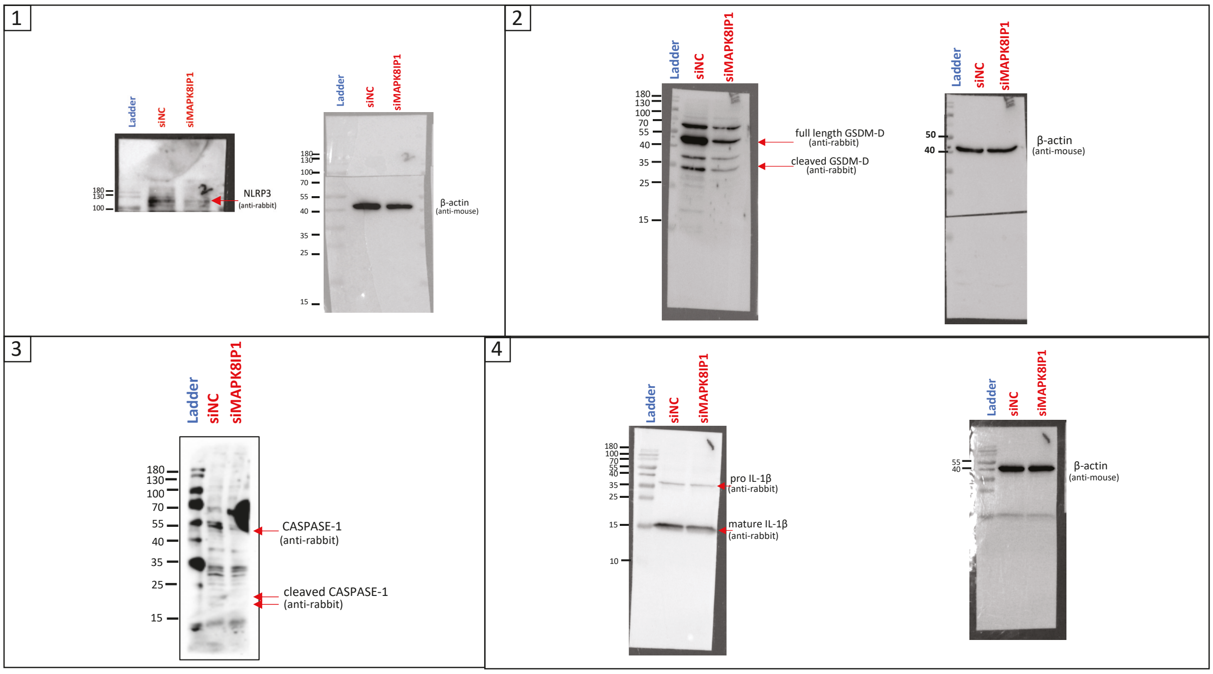

Figure S3. (replicate 3) The full-length western blot expressions after *Mapk8ip1* silencing. Western blot was conducted for **(1)** NLRP3, **(2)** GSDM-D (full length and cleaved N-terminal fragment), (**3)** CASPASE-1 (pro-caspase-1 and active cleaved caspase-1) and (**4)** IL-1β (pro and mature IL-1β). β-actin was used as endogenous control.

Figure S4 (replicate 4):

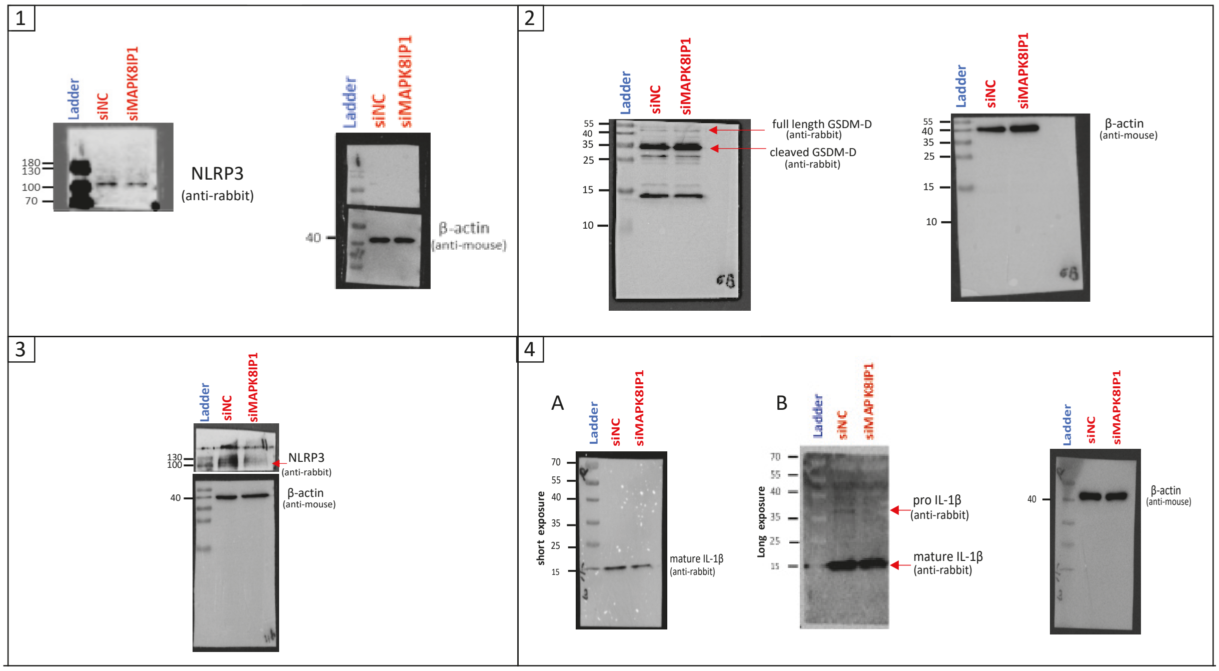

Figure S4. (replicate 4) The full-length western blot expressions after *Mapk8ip1* silencing. Western blot was conducted for **(1)** NLRP3, **(2)** GSDM-D (full length and cleaved N-terminal fragment), (**3**)NLRP3 and (**4)** IL-1β (pro and mature IL-1β) after (A) short exposure time and (B) long exposure time. β-actin was used as endogenous control.

Figure S5 (replicate 1):

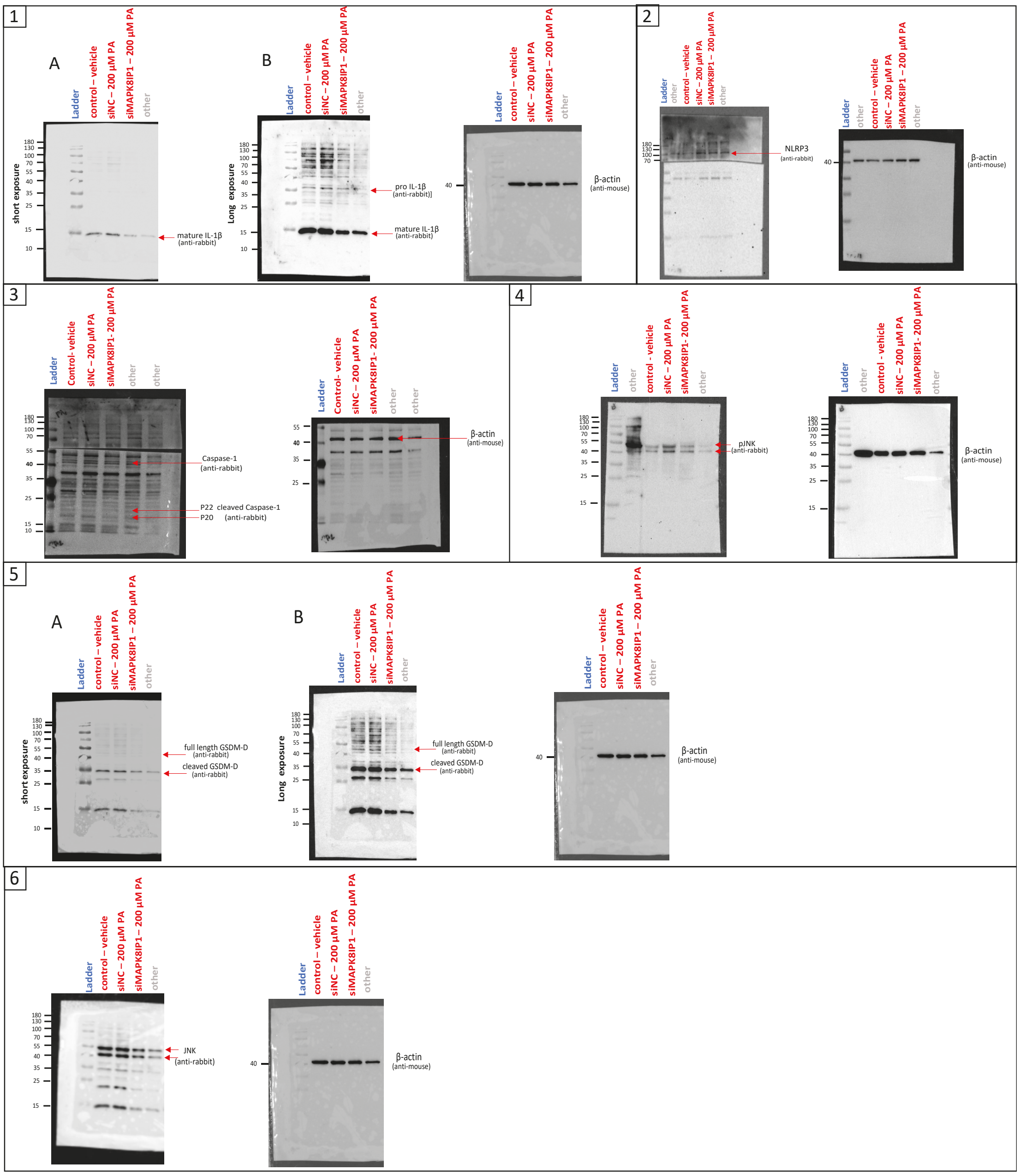

Figure S5. (replicate 1) The full-length western blot expressions showing the impact of *Mapk8ip1* silencing on inflammasome activation using LPS/PA-BSA. Western blot was conducted for **(1)** IL-1β (pro and mature IL-1β) after (A) short exposure time and (B) long exposure time, **(2)** NLRP3, **(3)** CASPASE-1 (pro-caspase-1 and active cleaved caspase-1), **(4)** pJNK, **(5)** GSDM-D (full length and cleaved N-terminal fragment) after (A) short exposure time and (B) long exposure time and **(6)** JNK. β-actin was used as endogenous control.

Figure S6 (replicate 2):

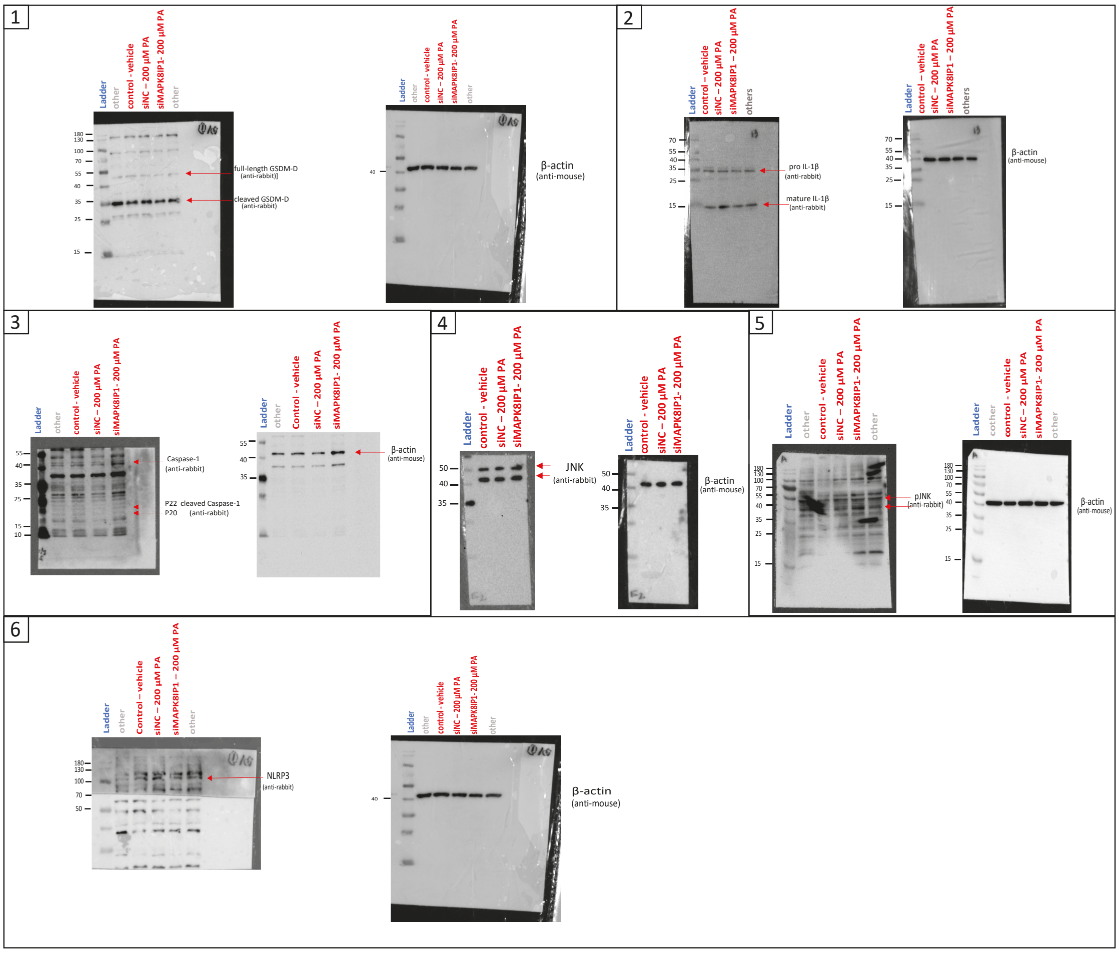

Figure S6. (replicate 2) The full-length western blot expressions showing the impact of *Mapk8ip1* silencing on inflammasome activation using LPS/PA-BSA. Western blot was conducted for **(1)** GSDM-D (full length and cleaved N-terminal fragment) **(2)** IL-1β (pro and mature IL-1β), (**3)** CASPASE-1 (pro-caspase-1 and active cleaved caspase-1), (**4)** JNK, **(5)** pJNK and **(6)** NLRP3. β-actin was used as endogenous control.

Figure S7 (replicate 3):

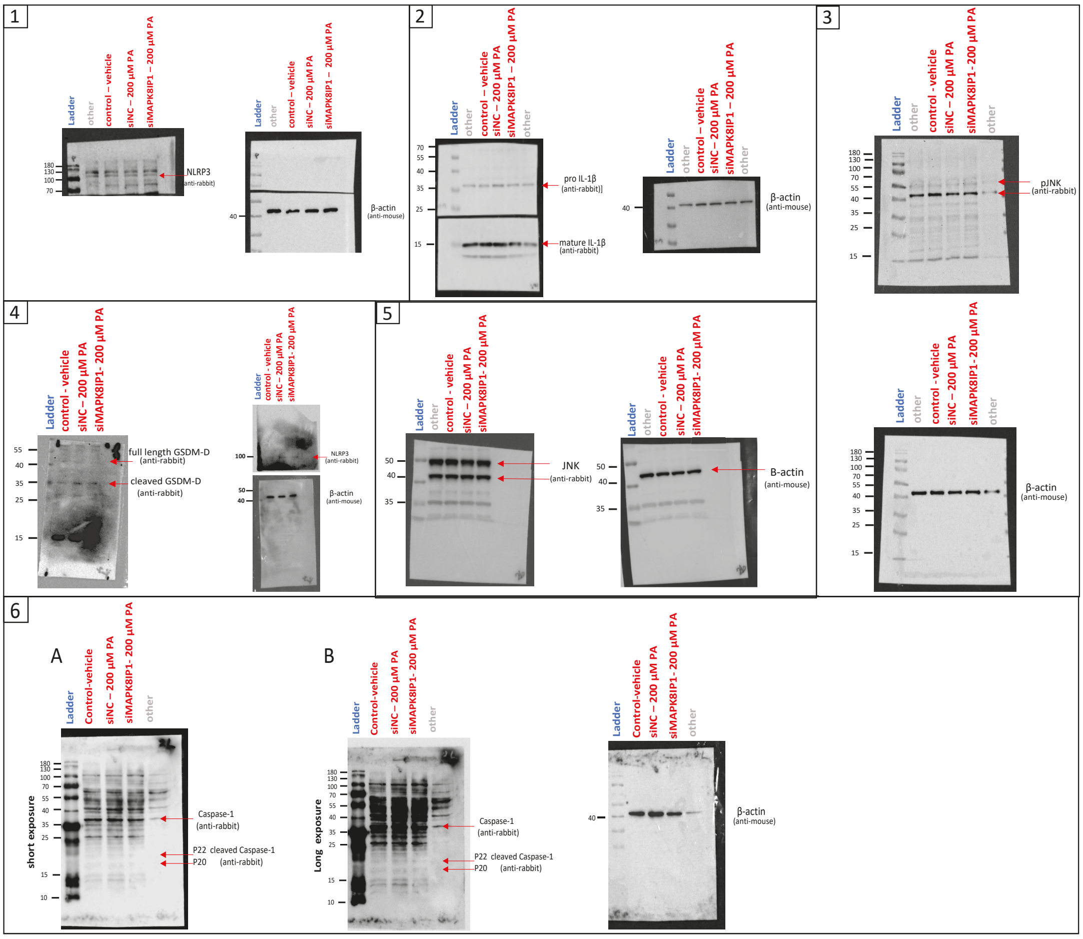

Figure S7. (replicate 3) The full-length western blot expressions showing the impact of *Mapk8ip1* silencing on inflammasome activation using LPS/PA-BSA. Western blot was conducted for **(1)** NLRP3, **(2)** IL-1β (pro and mature IL-1β), **(3)** pJNK, **(4)** GSDM-D (full length and cleaved N-terminal fragment) and NLRP3, **(5)** JNK and **(6)** CASPASE-1 (pro-caspase-1 and active cleaved caspase-1) after (A) short exposure time and (B) long exposure time. β-actin was used as endogenous control.
